# Quantifying variation across 16S rRNA gene sequencing runs in human microbiome studies

**DOI:** 10.1007/s00253-024-13198-z

**Published:** 2024-06-08

**Authors:** Andrew J. Hoisington, Christopher E. Stamper, Joseph C. Ellis, Christopher A. Lowry, Lisa A. Brenner

**Affiliations:** 1 Veterans Health Administration, Rocky Mountain Mental Illness Research Education and Clinical Center (MIRECC) for Veteran Suicide Prevention, Rocky Mountain Regional Veterans Affairs Medical Center (RMRVAMC), Aurora, CO USA; 2 Department of Physical Medicine and Rehabilitation, University of Colorado Anschutz Medical Campus, Aurora, CO USA; 3 Military and Veteran Microbiome: Consortium for Research and Education (MVM-CoRE), Aurora, CO USA; 4 Department of Systems Engineering and Management, Air Force Institute of Technology, Wright-Patterson Air Force Base, Dayton, OH USA; 5 Netellis LLC, Knoxville, TN USA; 6 Department of Integrative Physiology, Center for Neuroscience, and Center for Microbial Exploration, University of Colorado Boulder, Boulder, CO USA; 7 Department of Psychiatry, University of Colorado Anschutz Medical Campus, Aurora, CO USA; 8 Department of Neurology, University of Colorado Anschutz Medical Campus, Aurora, CO USA

**Keywords:** Microbiome, Sequencing variance, Accuracy, Precision, Positive controls

## Abstract

**Abstract:**

Recent microbiome research has incorporated a higher number of samples through more participants in a study, longitudinal studies, and metanalysis between studies. Physical limitations in a sequencing machine can result in samples spread across sequencing runs. Here we present the results of sequencing nearly 1000 16S rRNA gene sequences in fecal (stabilized and swab) and oral (swab) samples from multiple human microbiome studies and positive controls that were conducted with identical standard operating procedures. Sequencing was performed in the same center across 18 different runs. The simplified mock community showed limitations in accuracy, while precision (e.g., technical variation) was robust for the mock community and actual human positive control samples. Technical variation was the lowest for stabilized fecal samples, followed by fecal swab samples, and then oral swab samples. The order of technical variation stability was inverse of DNA concentrations (e.g., highest in stabilized fecal samples), highlighting the importance of DNA concentration in reproducibility and urging caution when analyzing low biomass samples. Coefficients of variation at the genus level also followed the same trend for lower variation with higher DNA concentrations. Technical variation across both sample types and the two human sampling locations was significantly less than the observed biological variation. Overall, this research providing comparisons between technical and biological variation, highlights the importance of using positive controls, and provides semi-quantified data to better understand variation introduced by sequencing runs.

**Key points:**

*• Mock community and positive control accuracy were lower than precision.*

*• Samples with lower DNA concentration had increased technical variation across sequencing runs.*

*• Biological variation was significantly higher than technical variation due to sequencing runs.*

**Supplementary Information:**

The online version contains supplementary material available at 10.1007/s00253-024-13198-z.

## Introduction

Microbiome research has grown exponentially as technical advances in sequencing and novel findings linking microbiome diversity and community structure to physiology and behavior encourage exploration in the field. Partially due to the rapid growth, there are challenges comparing results across studies due to differences in study design, cohorts of interest, sequencing technology, and/or sampling and analysis procedures. Initial standardization of sequencing methods emerged from standard operating procedures developed for major studies (e.g., Human Microbiome Project (Turnbaugh et al. 2007), Earth Microbiome Project (Gilbert et al. 2014), American Gut Project (McDonald et al. 2018). More recently, concentrated efforts of multiple research consortia have joined together (e.g., Microbiome Quality Control Project (Sinha et al. 2015), International Human Microbiome Standards (Cardona et al. 2012; Santiago et al. 2014), with government agencies (e.g., National Institute for Biological Standards and Controls, National Institute of Standards and Technology) to propose standards for conducting microbiome research. Moreover, the reporting of methods and data in microbiome studies has been aided by the introduction of the Strengthening the Organization and Reporting of Microbiome Studies (STORM) checklist (Mirzayi et al. 2021).

Researchers using the 16S rRNA gene for identification of bacteria in human samples have several key decision points that can impact findings. Pre-sequencing decisions include sampling methods (Sinha et al. 2016; Vogtmann et al. 2017), sample storage (Cardona et al. 2012), extraction kit (Kennedy et al. 2014), and primer selection (Abellan-Schneyder et al. 2021). In addition, over a dozen sequencing platforms and bioinformatics pipelines for analysis of the gut microbiome composition when using 16S rRNA amplicon sequencing are available, which introduces other biases to findings (Allali et al. 2017). Even sequencing platforms developed by the same company (e.g., Illumina MiSeq and iSeq, San Diego, CA, USA) have non-uniform sequencing outputs (Salamon et al. 2022).

One aspect of variability in microbiome sequencing that is less studied is technical reproducibility when using the same sequencing machine for multiple sequencing runs. A physical limitation of sequencing machines is the number of samples in each run to maintain adequate sequencing depth per sample. Studies that require multiple sequencing runs are common due to decreases in sampling costs, increases in desired samples per study, and the use of longitudinal studies. The aim of this study was to investigate the influence of sequencing runs on accuracy and precision, explore the use of positive controls, and increase understanding regarding the difference between technical (i.e., variation due to processes) and biological variation (i.e., variation due to different participants or same participant at a different time). To achieve these aims, we analyzed 995 16S rRNA gene sample sequencing results from multiple studies and positive controls with the same standard operating procedure.

## Materials and methods

The Military and Veterans Microbiome Consortium for Research and Education (MVM-CoRE, https://www.mirecc.va.gov/visn19/mvm/) housed within the Rocky Mountain Mental Illness Research Education and Clinical Center (MIRECC) for Veteran Suicide Prevention has been studying the gut, oral, and skin microbiome using 16S rRNA gene sequencing from 2016 to the present using Illumina MiSeq machines. Sequencing runs for this manuscript were from a longitudinal study of United States Veterans (study: US-VMP) and a longitudinal study of non-Veterans seeking Emergency Department (ED) care for a recent mild traumatic brain injury (TBI) (study: ED-TBI). Longitudinal studies enabled comparisons between samples from the same and other participants. Microbiome sample collection was the same procedure as outlined in the US-VMP study (Brenner et al. 2018), with the addition of the OmniGene Gut kit (Cat. No. OMR-200, DNA Genotek, Ottawa, Canada). Briefly, oral microbiome samples were self-collected with double-tipped polyurethane swabs (BD BBL™ CultureSwab™ EZ II, Cat. No. B220144, Fisher Scientific, Pittsburgh, PA, USA) from the buccal mucosa. Participants provided two fecal microbiome samples from the same bowel movement. One fecal microbiome sample was collected with a sterile dual tipped swab using the “first wipe” method and another sample used the OmniGene Gut kit. Fecal samples were collected in-person during a study visit and immediately frozen or at the participant’s residence and shipped to the Rocky Mountain MIRECC via standard ground shipping. Positive controls were the same DNA extracted from a pooled sample of two individuals using the same sample collection procedures as outlined for the US-VMP and the ED-TBI studies. Specifically, we collected fecal swab (“Positive Control Fecal Swab”), OmniGene (“Positive Control Fecal Omni”), and oral swab (“Positive Control Oral Swab”) from the same two participants and the same bowel movements for fecal samples. DNA was also extracted from a mock community microbial standards commercial kit (ZymoBiomics Microbial Community Standard, Cat. No. D6300, Zymo Research, Irvine, CA, USA). Identical procedures were followed for the sample collection and DNA extraction between the studies, positive controls, and the mock community.

Sample DNA was extracted from microbiome samples using the PowerSoil DNA extraction kit (Cat. No. 12955-4, Qiagen, Valencia, CA, USA) and quantified via Quant-IT dsDNA Assay Kit in triplicate (Cat. No. Q33120, Invitrogen, Waltham, MA, USA). DNA was extracted with 100 µL of C6 in the final step of the PowerSoil kit. Mock and positive controls were vortexed and pipette mixed prior to being aliquoted to ensure samples each had one freeze-thaw cycle. Marker genes in isolated DNA were polymerase chain reaction (PCR)-amplified using GoTaq Master Mix (Cat. No. M5133, Promega, Madison, WI, USA) and 515 F (5′-GTGCCAGCMGCCGCGGTAA-3′), 806 R (5′-GGACTACHVGGGTWTCTAAT-3′) primer pair (Integrated DNA Technologies, Coralville, IA, USA) targeting the V4 hypervariable region of the 16S rRNA gene modified with a unique 12-base sequence identifier for each sample and the Illumina adapter (Caporaso et al. 2012). The thermal cycling program consisted of an initial step at 94 °C for 3 min followed by 35 cycles (94 °C for 45 s, 55 °C for 1 min, and 72 °C for 1.5 min), and a final extension at 72 °C for 10 min. Products from the duplicate PCR reactions were pooled and successful amplification was visualized on an agarose gel. PCR products were cleaned, normalized, and sequenced at a university sequencing center on an Illumina MiSeq using V2 chemistry and 300 cycle, 2 × 150-bp paired end sequencing. All sequencing was conducted between October 2022 and August 2023. The sequencing center was not involved in the study design or manuscript preparation. Demultiplexed single-end sequences were deposited in the NCBI Sequence Read Archive (BioProject accession ID: PRJNA1101562).

Sequencing data were initially processed using the Quantitative Insights Into Microbial Ecology program (QIIME2 v. 2023.5) (Bolyen et al. 2019). The Deblur algorithm (Amir et al. 2017) was used to denoise demultiplexed sequences. Quality-filtered sequences were assigned taxonomic classification based on the Silva database (v. 138) (Quast et al. 2012). Mock community and positive controls samples were rarefied to 8,600 sequences per sample and participant samples were rarefied at 11,000 sequences per sample.

Statistical analyses were performed with QIIME2 and the open-source statistical software R v. 4.2.2 (The R Core Team 2020) (https://www.R-project.org). All statistical tests were conducted with a two-tailed alpha level of 0.05. The alpha diversity metrics assessed were Observed Amplicon Sequencing Variants (ASVs), Shannon Diversity Index, and Pielou’s Evenness. Beta diversity was performed using the vegan package (Oksanen et al. 2008) for unweighted UniFrac and weighted UniFrac (Lozupone et al. 2006). Statistical differences for sequencing runs were calculated through pairwise permutational multivariate analysis of variance (PERMANOVA) with 10,000 permutations with the “adonis2” function. Microbial measures of taxonomic relative abundance were aggregated at the genus level. Intraclass correlation coefficient (ICC) is a measure of reliability or reproducibility that can be used to quantify the biological variability. To calculate ICC, genus or alpha diversity indices were first normalized with the bestNormalize function (Peterson 2021). Repeatability estimation of ICC was with a generalized linear mixed-effect model fitted by restricting maximum likelihood in a Gaussian datatype with 1000 bootstraps and permutations (Stoffel et al. 2017). ICC values range from 0 (i.e., no stability) to 1 (i.e., perfect stability). Values of ICC above 0.5 were considered high microbiome stability (Bobak et al. 2018). ICC was only calculated for mock community based on the number of repeated samples within the runs. Stability was also evaluated through assessment of percent coefficient of variation (%CV) for alpha diversity and for genera that had a mean relative abundance over 1% (i.e., “most abundant genera”).

## Results

### Accuracy and precision in simplified (mock) microbial communities

**Fig. 1:**
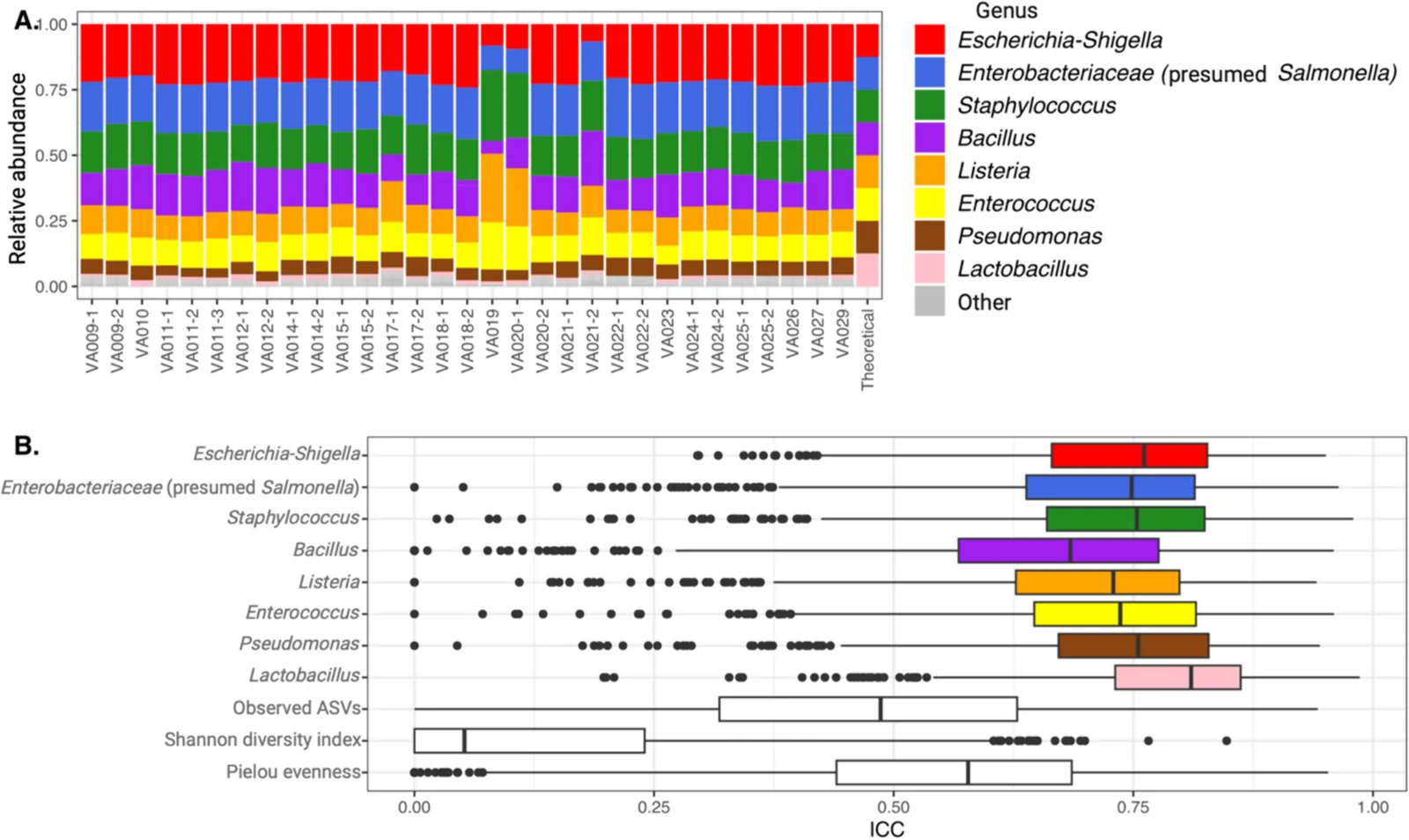
Mock community relative abundance (%) for all samples and theoretical values based on even distribution of the eight genera (run is denoted by VA, data with multiple samples in the same run are denoted by a dash) (**A**) and intraclass correlation coefficients for eight genera and three diversity indexes (coefficient of variations for most abundant taxa and alpha diversity measures in Supplemental Table S1) (**B**)

The mock community with a DNA concentration of 11.5 ng/µL was sequenced 31 times in 18 sequencing runs. Eight ASVs, corresponding with the expected number of mock community taxa, were observed across all samples, representing 97.1% relative abundance (i.e., Total False Positive Relative Abundance (Amos et al. 2020) = 2.9%). The mock community was expected to have evenly distributed taxa with 12.5% relative abundance per taxa. We observed an increased relative abundance of *Escherichia-Shigella* (mean 20.5% ± standard deviation 4.4%), *Enterobacteriaceae* (18.2% ± 2.8%), and *Staphylococcus* (16.4% ± 2.9%), while underrepresented taxa included *Pseudomonas* (5.4% ± 1.6%) and *Lactobacillus* (1.7% ± 0.4%) (Fig. 1A, Supplemental Table S1). Despite the variance in relative abundance to theoretical values, all eight taxa had ICC values in the high stability range (Fig. 1B). Observed ASVs (58.9 ± 23.9) were higher in the mock community than the expected number of eight, yet lower than the positive controls revealing this sample type is less diverse (Supplemental Fig. S1).

### Precision in complex (positive control) microbial communities

**Fig. 2:**
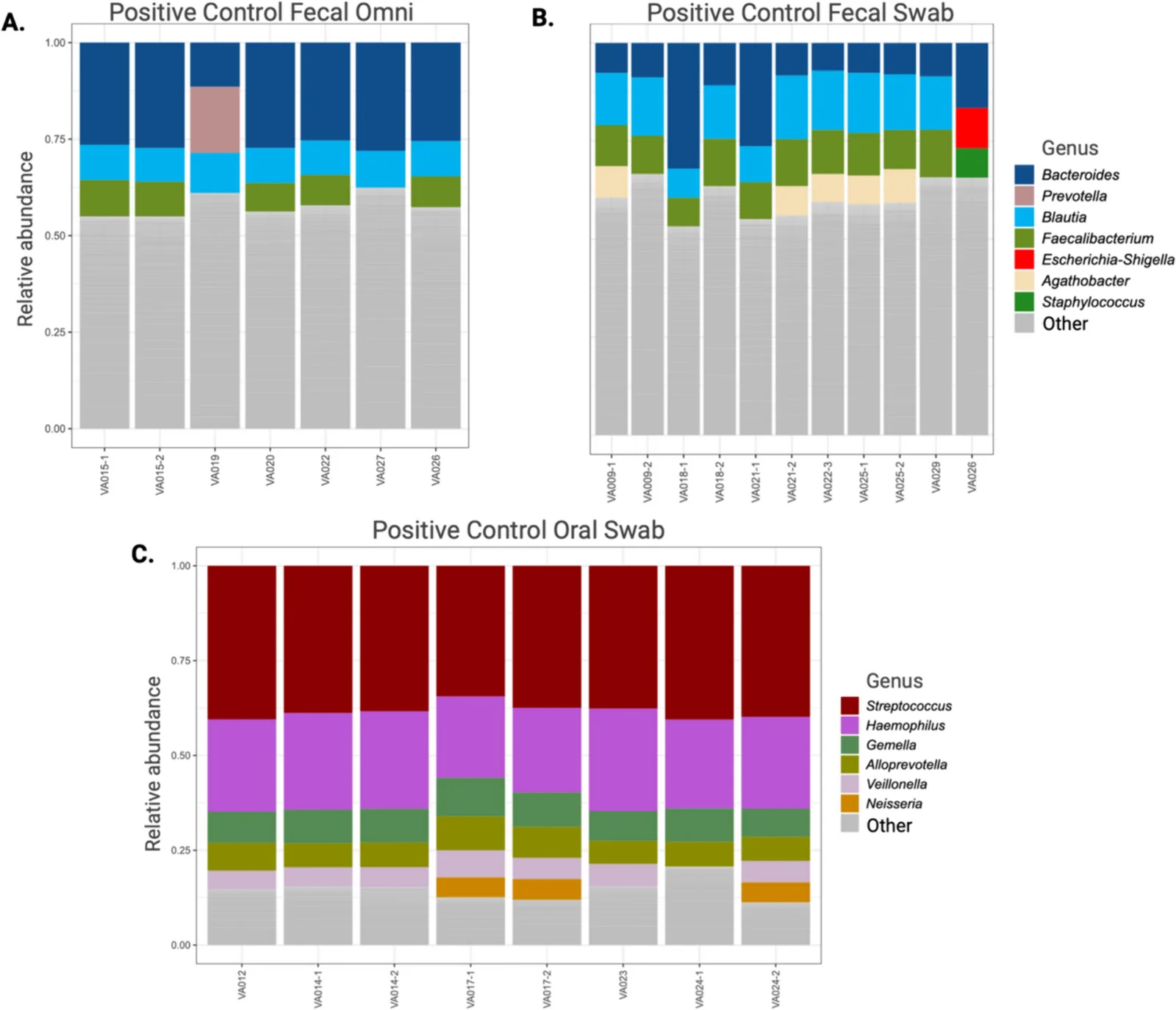
Genera with the highest relative abundances for positive control samples from Positive Control Fecal Omni (**A**); Positive Control Fecal Swab (**B**); Positive Control Oral Swab (**C**) run denoted by VA and data with multiple samples in the same run are denoted by a dash; additional details on taxonomic abundance provided in Supplemental Tables S2–4)

The Positive Control Fecal Omni sample yielded a DNA concentration of 45.7 ng/µL and was sequenced seven times across six runs. The most prevalent genera were *Bacteroides* (24.5% ± 5.9%), *Blautia* (9.3% ± 0.6%), *Faecalibacterium* (7.3% ± 2.2%), and *Prevotella* (6.1% ± 5.0%) (Fig. 2A). The mean measured alpha diversity values in the Positive Control Fecal Omni samples were 221 ± 22.0 for Observed ASVs, 4.2 ± 0.1 for Shannon diversity index, and 0.78 ± 0.02 for Pielou’s evenness. The mean %CV for the most abundant genera (1% or higher, *n* = 20) was 40.0% (range 6.1–81.1%), significantly lower compared to the ED-TBI Omni participant samples (*t*-test, *p* < 0.001; mean 141.8%, range 59.3–257.8%) (Supplemental Table S2).

Seven runs were conducted with eleven samples from the Positive Control Fecal Swab sample at a DNA concentration of 35.5 ng/µL. The most abundant genera observed were *Bacteroides* (12.8% ± 8.6%), *Blautia* (12.5% ± 3.7%), *Faecalibacterium* (10.0% ± 2.1%), and *Agathobacter* (5.9% ± 2.4%) (Fig. 2B). The samples had a mean of 219 observed ASVs per sample (± 25.9), Shannon diversity index of 4.22 (± 0.06), and Pielou’s evenness of 0.79 (± 0.03). The mean %CV for the most abundant genera (*n* = 22) was 36.0% (range 13.6–68.5%), significantly lower than the US-VMP Fecal Swab participant samples (*t*-test, *p* < 0.001; mean 215.8%, range 98.8–523.2%) **(**Supplemental Table S3**)**.

Eleven Positive Control Oral Swab samples at a DNA concentration of 8.7 ng/µL were sequenced in five runs. The most abundant genera observed in the Positive Control Oral Swab were *Streptococcus* (38.4% ± 2.0%), *Haemophilus* (24.2% ± 1.8%), and *Gemella* (8.7% ± 0.1%) (Fig. 2C). Alpha diversity values—all of which were lower than Positive Control Fecal Swab and Positive Control Fecal Omni samples—were measured for Shannon diversity index (2.2 ± 0.1), Observed ASV (60.6 ± 13.0), and Pielou’s evenness (0.5 ± 0.03). The mean %CV for the most abundant genera (*n* = 9) was 11.0% (range 5.3–17.2%), significantly lower than ED-TBI Oral Swab participant samples (*t*-test, *p* = 0.023; mean 218.6%, range 42.1–787.9%) **(**Supplemental Table S4**)**.

All positive controls were analyzed to compare alpha and beta diversity trends. Beta diversity among the positive control types was significantly different using either Weighted UniFrac (PERMANOVA, *p* = 0.001) or Unweighted UniFrac (PERMANOVA, *p* = 0.001) (Fig. S2). All pairwise comparisons were significantly different for Weighted and Unweighted UniFrac (i.e., *p* < 0.05), including Positive Control Fecal Omni and Positive Control Fecal Swab samples. Three calculated alpha diversity metrics were significantly different among the positive control sample types (Kruskal–Wallis Rank Sum Test, *p* < 0.001) (Supplemental Fig. S1). Alpha diversities were not different between Positive Control Fecal Omni and Positive Control Fecal Swab samples (*t*-test: Observed ASVs, *p* = 0.75, Shannon Diversity Index *p* = 0.26, Pielou’s Evenness, *p* = 0.22).

### Precision in participant (longitudinal) microbial communities

**Fig. 3:**
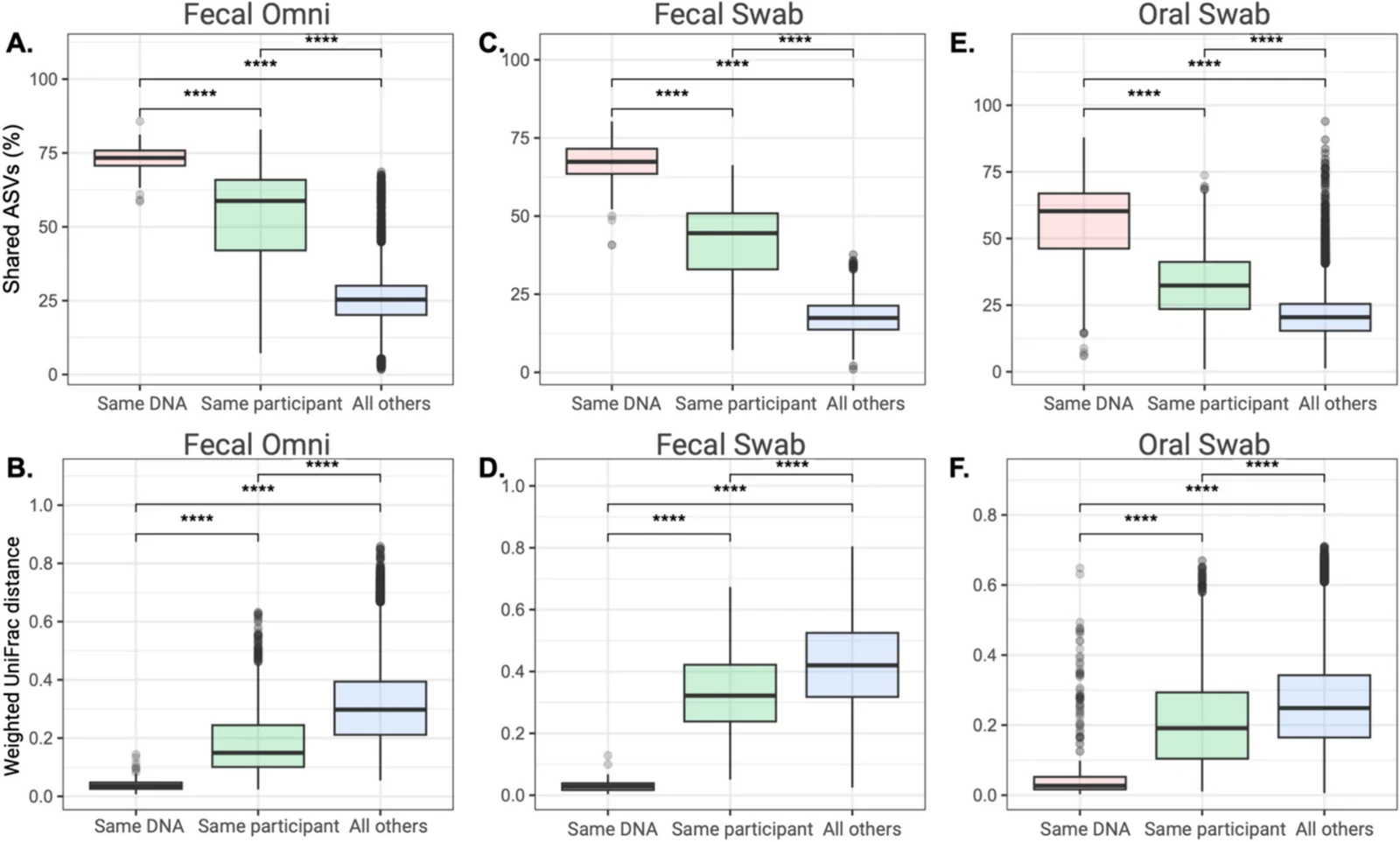
Comparison based on either same participant sample in two different sequencing runs (Same DNA), same participant at different times from different sequencing runs, and remaining samples compared (all others) for as follows: Shared ASVs in ED-TBI Fecal Omni samples (**A**); Weighted UniFrac distance in ED-TBI Fecal Omni samples (**B**); Shared ASVs in US-VMP Fecal Swab samples (**C**); Weighted UniFrac distance in US-VMP Fecal Swab samples (**D**); Shared ASVs in ED-TBI Oral Swab samples (**E**); Weighted UniFrac distance in ED-TBI Oral Swab samples (**F**). (****p < 0.001)

Two longitudinal microbiome studies enabled comparison across sequencing runs with repeated samples from the same participants. The fecal omni ED-TBI participant samples included 154 paired samples (i.e., exact same DNA sequenced twice) across six sequencing runs. The fecal swab US-VMP participant samples included 63 paired samples across five sequencing runs. Finally, the Oral Swab participant samples included 252 paired samples across four sequencing runs. Extracted DNA concentration was highest for fecal Omni samples (24.9 ± 19.1 ng/µL), followed by US-VMP Fecal Swab samples (12.0 ± 15.0 ng/µL) and Oral Swab samples (4.7 ± 6.8 ng/µL) (Supplemental Fig. S3). For all three sample types, participant microbial communities were significantly different based on Weighted UniFrac (PERMANOVA, *p* < 0.001). The microbial communities in Oral Swab samples were also significantly different based on run (PERMANOVA, *p* = 0.008), while ED-TBI Fecal Omni and US-VMP Fecal Swab communities were not significantly different (PERMANOVA, *p* = 0.36 and *p* = 0.34). Paired samples shared the most ASVs in the ED-TBI Fecal Omni samples, then US-VMP Fecal Swab samples, and finally ED-TBI Oral Swab samples (Fig. 3A, C, E). The same trend was observed in Weighted UniFrac distances (Fig. 3B, D, F). In all three sample types, the paired samples shared the most ASVs and had the most similar microbial community structure compared to samples from the same participant or other participants in the study.

## Discussion

Accuracy is the agreement between a measured value and the items true value (Budowle et al. 2014) and was evaluated via an eight evenly distributed genera mock community standard. In the oversimplified mock community, genera were both over-represented (e.g., *Escherichia-Shigella* and *Salmonella*) and under-represented (e.g., *Pseudomonas* and *Lactobacillus*), potentially influenced by primer selection, extraction kit, sequencing machine, and bioinformatics pipeline (for more information see Karstens et al. (2021) or Abellan–Schneyder et al. (2021). The limited accuracy observed when sequencing mock communities is unsurprising based on previous findings (Fouhy et al. 2016; Yeh et al. 2018); however, this concerning issue is outside the scope of this manuscript. Advancements in mock communities (Mori et al. 2023) have increased the complexity (i.e., number of taxa) and added a focused target (i.e., taxa related to study area microbiome) that should improve accuracy in the future. Precision is the degree to which repeated measurements return the same results (Budowle et al. 2014). The simplified mock community had high stability across all eight genera and the microbial community. Precision was less stable in alpha diversity, perhaps due to the relatively small abundance of ASVs that had exaggerated impacts on these measures.

Precision was also assessed in more complex communities through sequencing the same DNA from positive control samples for fecal Omni, fecal swab, and oral swab samples. Assessment of fecal Omni and fecal swab positive controls had differing stability across sequencing runs with fecal Omni showing greater precision in taxonomic measures and alpha diversity in comparison to fecal swabs. Generally, the use of positive controls in microbiome studies has been low, recorded in under 10% of research up to 2018 (Hornung et al. 2019), yet the use of positive controls is expected to increase with the introduction of multiple commercially available positive controls and literature on the subject. If precision (and therefore reliability) is of importance to a microbiome study or laboratory, the continual use of the same DNA across time is invaluable. We recommend the use of a pooled positive control sample from the same biogeographical region of interest and input DNA concentration in all sequencing runs to accurately assess sequencing precision and provide quality control metrics to decide acceptability of individual sequencing runs.

Fecal Omni samples and fecal swab samples taken from the same bowel movement and the same individuals had differing microbial communities, indicating either sample processing, DNA concentration, or another factor is important in stability across sequencing runs. We used participant samples across sequencing runs to assess the impact of DNA concentration on precision. The highest stability was observed for fecal Omni samples, followed by fecal swab samples, and then oral swab samples. DNA concentrations followed the same order with the highest concentrations in fecal Omni samples. The ability to sequence low biomass accurately is an important topic in microbiome research (Bender et al. 2018) with variability in precision introduced from exogenous bacterial DNA concentrations (Salter et al. 2014) that can be amplified through select bioinformatics pipelines (Caruso et al. 2019). Importantly in the present study for all sample types, the technical variation observed between sequencing runs was significantly lower than the biological variance of repeated samples from the same individual or other participants. Therefore, while variance due to sequencing runs should be assessed, the differences at a microbial community level appear to be a minor issue in clinical research.

Clinical studies of microbial communities have recently focused less on community measures (e.g., lower alpha diversity results in worse health outcomes) and more on individual taxa variations between timepoints or participant cohorts. Our results suggest care should be taken when reporting differences in taxa relative abundance from a 16S rRNA gene sequencing study across sampling runs. The most abundant taxa in our mock community and positive controls had %CVs across the sequencing runs from 5.3% to 81.1%, similar in magnitude that others have reported in microbiome quality control studies (Barlow et al. 2020; Bender et al. 2018). Although concerning, the observed %CVs for genus-level taxa are similar in magnitude to other historically used biological measurements across platforms for blood-based chemokines and cytokines (McKay et al. 2017). Microbiome analysis with ICC to determine stability is a recently used statistical approach in the field, first appearing in a 2017 manuscript on wild red squirrels (Ren et al. 2017). ICC revealed that all eight genera in the mock community were stable. While the %CV at the genus level were variable across sequencing runs, their values were generally 75% less that %CV across studies again indicating biological variance dominates technical variance. However, technical variance at a genus level might still be an important factor when applying one of the many differentially abundant estimation tools that are often used in clinical research.

The present study has limitations including that the results were only obtained from one sequencing center over the period of 1 year. It is possible that the use of other sequencing centers or sequencing across a longer period of time could have different levels of consistency between sequencing runs compared to what we observed. Another limitation was the use of a simplified mock community with only eight bacterial genera. More recently developed mock communities have additional complexity and new protocols exist to assist laboratories in developing a study-specific mock community (Colovas et al. 2022). A strength of this study was the large sample size that included multiple positive controls, two sampling methods, and two human body locations. Additionally, the use of longitudinal samples enabled direct comparison of technical and biological variation.

In conclusion, this study investigated the technical variability introduced between sequencing runs on the resulting taxonomy, alpha diversity, and beta diversity. Specifically, we characterized the variability with a simplified mock community, positive control samples, and actual participant samples for two sampling methods (e.g., swab or commercially available stabilization kits) and two human sampling locations (i.e., fecal and oral). Based on our results, the following are recommendations for laboratories to understand and limit variation across sequencing runs: (1) use positive controls from the same biogeographic region in each sequencing run to assess variation; (2) consider more complex positive controls when feasible; (3) use a standardized stabilizing agent in sample collection; and (4) normalize DNA concentrations pre-amplification. Given the number of sequencing studies that exist to date, the development of bioinformatics tools to adequately adjust results post sequencing is an important knowledge gap. These results provide a context for technical variability in microbiome studies that span multiple sequencing runs, between studies from the same laboratory, and between laboratories that use identical standard operating procedures. Additionally, results provide a context to establish meaningful biological variances that are not attributed to technical variance that can be used to verify adequate sequencing run quality or adjust power estimates for sample size calculations.

## Electronic supplementary material

Below is the link to the electronic supplementary material.

Supplementary Material 1

## Data Availability

Demultiplexed single-end sequences were deposited in the NCBI Sequence Read Archive (BioProject accession ID: PRJNA1101562).

## Declarations

### Disclaimer

The views, opinions, and/or findings contained in this article are those of the author(s) and should not be construed as an official Department of Defense or VA position, policy, or decision unless so designated by other documentation.
