# Supplementary material for: Quantifying variation across 16S rRNA gene sequencing runs in human microbiome studies
Source: Appl Microbiol Biotechnol. 2024 Jun 8;108:367. doi: 10.1007/s00253-024-13198-z (PMC11162379; doi:10.1007/s00253-024-13198-z)
Supplement: Supplementary file 1 [file 253_2024_13198_MOESM1_ESM.pdf]

Journal: *Applied Microbiology and Biotechnology*

Quantifying Variation Across 16S rRNA Gene Sequencing Runs in Human Microbiome Studies

Andrew J. Hoisington[1-4*], Christopher E. Stamper[1-3], Joseph C. Ellis[5], Christopher A. Lowry[1-3,6], Lisa A. Brenner[1-3,7,8]

[1]Veterans Health Administration, Rocky Mountain Mental Illness Research Education and Clinical Center (MIRECC) for Veteran Suicide Prevention, Rocky Mountain Regional Veterans Affairs Medical Center (RMRVAMC), Aurora, Colorado, United States of America

[2]Department of Physical Medicine and Rehabilitation, University of Colorado Anschutz Medical Campus, Aurora, Colorado, United States of America

[3]Military and Veteran Microbiome: Consortium for Research and Education (MVM-CoRE), Aurora, Colorado, United States of America

[4]Department of Systems Engineering and Management, Air Force Institute of Technology, Wright-Patterson Air Force Base, Ohio, United States of America

[5]Netellis LLC, Knoxville, TN, United States of America

[6]Department of Integrative Physiology, Center for Neuroscience, and Center for Microbial Exploration, University of Colorado Boulder, Boulder, Colorado, United States of America

[7]Department of Psychiatry, University of Colorado Anschutz Medical Campus, Aurora, CO, United States of America

[8]Department of Neurology, University of Colorado Anschutz Medical Campus, Aurora, Colorado, United States of America

*Corresponding author

Andrew Hoisington

Email: andrew.hoisington@va.gov

Supplemental Table S1. Mock community genus and diversity index coefficients of variation

Genus	Relative abundance (mean ± standard deviation, %)	Coefficient of variation (%)
Escherichia-Shigella	20.4 ± 4.4	21.4
Enterobacteriaceae (presumed *Salmonella*)	18.2 ± 2.8	15.4
Staphylococcus	16.4 ± 2.9	18.6
Bacillus	13.1 ± 4.1	22.0
Listeria	11.0 ± 3.8	34.6
Enterococcus	10.6 ± 2.1	19.9
Pseudomonas	5.4 ± 1.0	18.6
Lactobacillus	1.7 ± 0.4	36.4

Diversity index	Value (mean ± standard deviation)	Coefficient of variation (%)
Observed ASVs	58.9 ± 23.9	40.6
Shannon diversity index	2.07 ± 0.09	4.2
Pielou evenness	0.54 ± 0.08	14.4

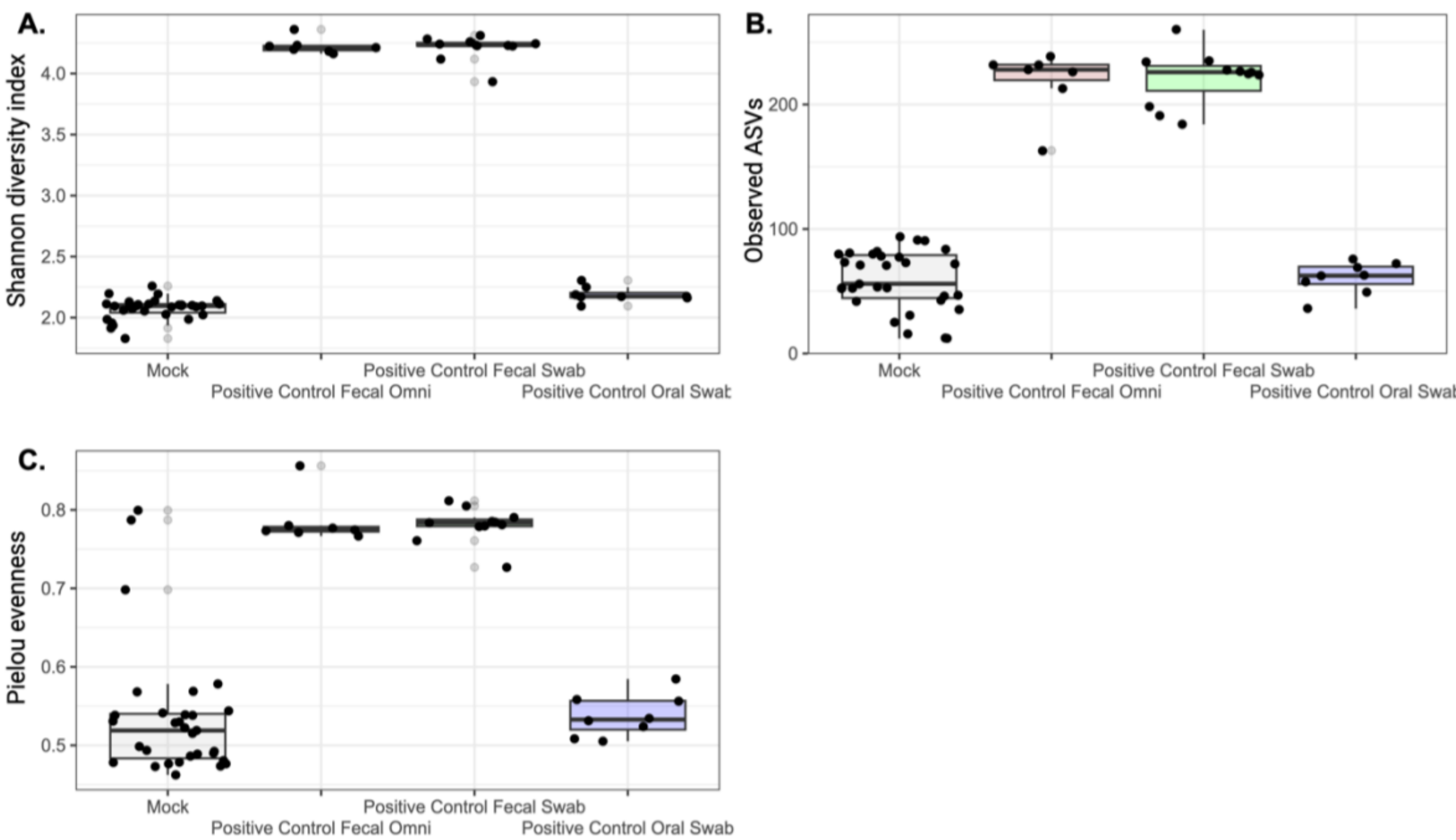

Supplemental Fig. S1. Positive Controls boxplots for (A) Shannon diversity index, (B) Observed ASVs, and (C) Pielou evenness

Supplemental Table S2. Positive Control Fecal Omni For genera relative abundance (over 1%) and coefficients of variation. Coefficients of variation from ED-TBI Fecal Omni participants for comparison.

**Genus**	**Positive Control Fecal Omni (mean ± standard deviation, %)**	**Positive Control Fecal Omni coefficient of variation (%)**	**ED-TBI Fecal Omni participants coefficient of variation (%)**
Bacteroides	24.5 ± 5.86	23.9	59.3
Blautia	9.3 ± 0.6	6.1	72.1
Faecalibacterium	7.3 ± 2.2	30.1	84.1
Prevotella	6.1 ± 4.9	81.1	197.1
Prevotellaceae_Ga6A1_group	4.7 ± 2.1	44.5	-
Subdoligranulum	3.1 ± 0.5	16.1	95.6
Agathobacter	3.0 ± 0.8	26.8	124.5
Clostridia_UCG-014	2.7 ± 1.0	38.5	257.8
Fusicatenibacter	2.2 ± 0.5	20.8	113.2
Coprococcus	2.0 ± 0.4	21.0	96.4
Prevotellaceae_NK3B31_group	2.0 ± 0.8	40.3	-
Parabacteroides	2.0 ± 0.4	18.9	113.0
Alistipes	1.8 ± 0.8	44.3	110.2
Dorea	1.5 ± 0.5	32.8	78.6
Christensenellaceae_R-7_group	1.4 ± 0.5	34.2	253.1
CAG-352	1.3 ± 0.6	45.1	207.4
Bifidobacterium	1.2 ± 0.9	71.8	163.6
[Eubacterium]_siraeum_group	1.1 ± 0.5	43.0	232.6
Ruminococcus	1.1 ± 0.6	55.4	145.6
Sutterella	1.0 ± 0.2	24.8	147.3

**Diversity index**	**Positive control Fecal Omni (mean ± standard deviation, %)**	**Positive control Fecal Omni coefficient of variation (%)**	**Fecal Omni all participants coefficient of variation (%)**
Observed ASVs	221 ± 26.1	11.8	33.5
Shannon diversity index	4.2 ± 0.06	1.5	17.6
Pielou evenness	0.8 ± 0.3	4	12.3

Supplemental Table S3. Positive Control Fecal Swab For genera mean relative abundance (over 1%) and coefficients of variation. Coefficients of variation from US-VMP Fecal Swab participants for comparison.

Genus	Positive Control Fecal Swab (mean ± standard deviation, %)	Positive Control Fecal Swab coefficient of variation (%)	US-VMP Fecal Swab participants coefficient of variation (%)
Bacteroides	12.9 ± 8.6	66.7	98.8
Blautia	12.5 ± 3.7	29.7	127.1
Faecalibacterium	10.0 ± 2.4	21.1	130.8
Agathobacter	5.9 ± 2.1	40.6	164.1
Subdoligranulum	4.5 ± 1.2	25.9	166.6
Prevotellaceae_Ga6A1_group	3.9 ± 0.5	13.6	-
Fusicatenibacter	3.3 ± 0.9	26.5	177.1
Dorea	2.9 ± 1.3	45.4	137.9
Coprococcus	2.9 ± 0.8	26.9	205.8
Clostridia_UCG-014	2.7 ± 0.4	16.1	409.0
Prevotella	2.0 ± 1.4	68.5	255.3
Anaerostipes	1.9 ± 0.8	41.4	126.0
CAG-352	1.8 ± 0.3	19.4	176.4
[Ruminococcus]_torques_group	1.6 ± 0.7	46.5	207.5
Bifidobacterium	1.5 ± 0.5	30.2	222.0
Prevotellaceae_NK3B31_group	1.3 ± 0.6	48.8	523.2
Lachnospiraceae_ND3007_group	1.3 ± 0.5	38.2	284.8
[Eubacterium]_hallii_group	1.3 ± 0.4	31.1	158.7
Christensenellaceae_R-7_group	1.2 ± 0.2	13.6	199.3
Erysipelotrichaceae_UCG-003	1.2 ± 0.5	41.5	310.3
Parabacteroides	1.1 ± 0.6	52.7	144.0
[Ruminococcus]_gauvreauii_group	1.1 ± 0.5	47.5	307.0

Diversity index	Positive Control Fecal Swab (mean ± standard deviation, %)	Positive Control Fecal Swab coefficient of variation (%)	US-VMP Fecal Swab participants coefficient of variation (%)
Observed ASVs	219 ± 21.7	9.9	42.7
Shannon diversity index	4.2 ± 0.1	2.5	31.2
Pielou evenness	0.8 ± 0.02	2.8	26.6

Supplemental Table S4. Positive Control Oral Swab For genera mean relative abundance (over 1%) and coefficients of variation. Coefficients of variation from ED-TBI Oral Swab participants for comparison.

Genus	Positive control Oral Swab (mean ± standard deviation, %)	Positive control Oral Swab coefficient of variation (%)	ED-TBI Oral Swab participants coefficient of variation (%)
Streptococcus	38.5 ± 2.0	5.3	42.1
Haemophilus	24.3 ± 1.8	7.6	130.7
Gemella	8.7 ± 0.8	9.6	70.9
Alloprevotella	7.0 ± 1.0	14.9	260.3
Veillonella	5.5 ± 0.7	13.6	141.9
Neisseria	5.0 ± 0.3	6.2	203.5
Granulicatella	2.1 ± 0.2	9.9	169.8
Streptobacillus	1.9 ± 0.3	14.4	787.9
Fusobacterium	1.0 ± 0.2	17.2	160.0

Diversity index	Positive control Oral Swab (mean ± standard deviation, %)	Positive control Oral Swab coefficient of variation (%)	ED-TBI Oral Swab participants coefficient of variation (%)
Observed ASVs	60.6 ± 13.0	21.5	57.9
Shannon diversity index	2.2 ± 0.1	2.9	39.5
Pielou evenness	0.5 ± 0.03	5.0	31.5

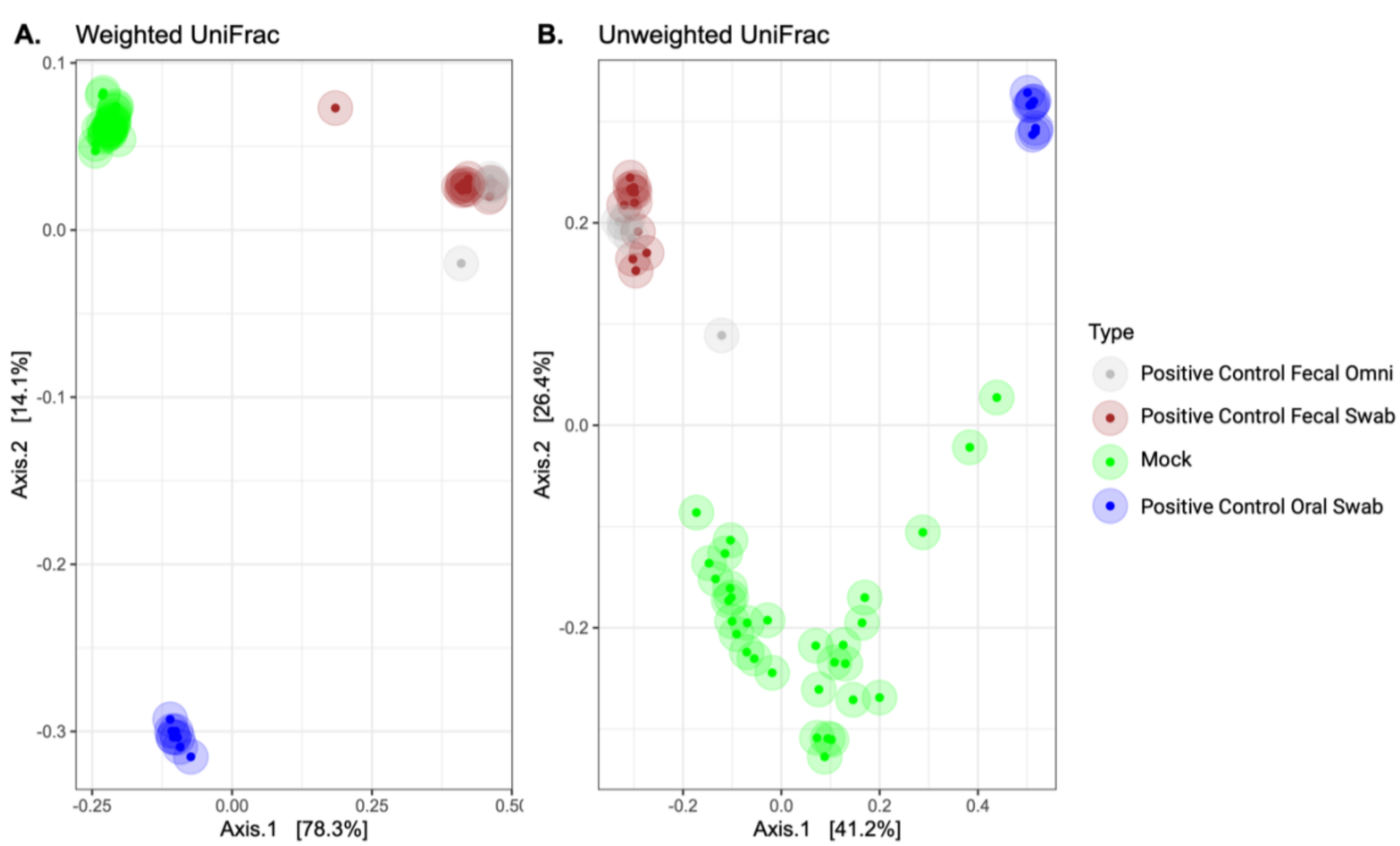

Supplemental Fig. S2. Positive Controls PCoA for (A) Weighted UniFrac, (B) Unweighted UniFrac.

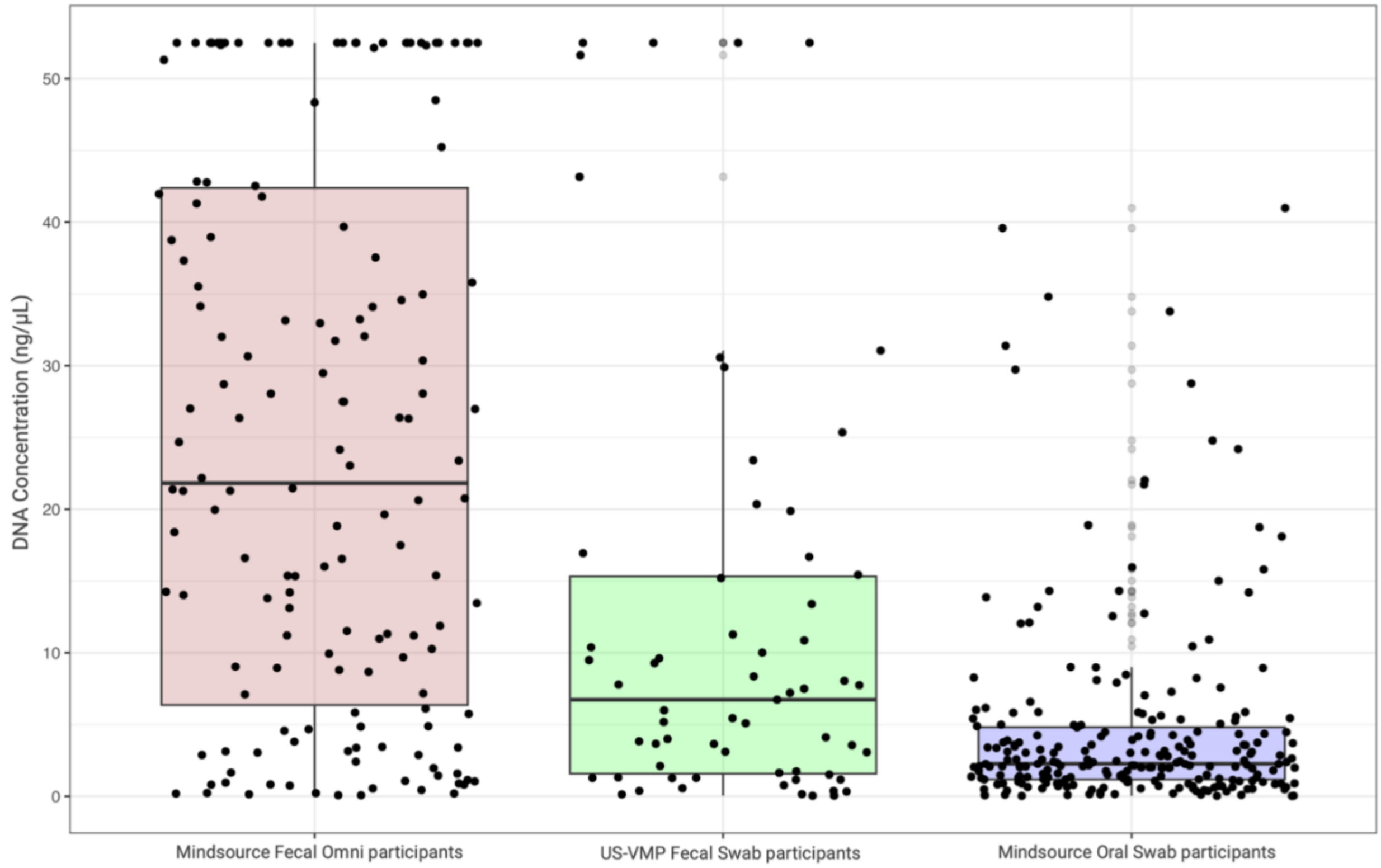

Supplemental Fig. S3. Extracted DNA concentrations from participant samples.
